# Transdermal Estradiol Patches for ADT in Locally Advanced Prostate Cancer

**DOI:** 10.1056/NEJMoa2511781

**Published:** 2026-03-25

**Authors:** Ruth E. Langley, Duncan C. Gilbert, Stephen Mangar, Stuart Rosen, Ellie Bourmaki, Hannah L Rush, Subramaniam Kananga Sundaram, Abdulla Alhasso, Roger Kockelbergh, Hoda Abdel-Aty, Claire L Amos, Louise Brown, Simon Brown, Charlene Carvalho, Kitty Chan, Gerald Collins, William Cross, John Deighan, Sanjay Dixit, Trinh Duong, James Dyer, Joanna Gale, Silke Gillessen, Anna Griffiths, Marc Laniado, Anna Lydon, Neil McPhail, Archie MacNair, Sanjeev Madaan, John Marshall, David Matheson, Robin Millman, Wael Mohamed, Laura Murphy, Krishna Narahari, Christopher Parker, Miguel Panades, Alvan Pope, Arpita Raval, Angus Robinson, Martin Russell, Christopher Scrase, Matthew Sydes, Rafal Turo, Ram Venkitaraman, Simona Wade, Howard Kynaston, Gerhardt Attard, Nicholas D. James, Noel Clarke, Mahesh K. Parmar, Matthew Nankivell

**Affiliations:** 1 MRC Clinical Trials Unit at University College London, Institute of Clinical Trials and Methodology, London UK; 2 Imperial College Healthcare NHS Trust, London, UK; 3 London Northwest and Royal Brompton Hospitals and Imperial College, London, UK; 4 Pinderfields Hospital Mid-Yorkshire Teaching NHS Trust, Wakefield UK; 5 The Beatson West of Scotland Cancer Centre, Glasgow, UK; 6 Department of Urology, University Hospitals of Leicester, Leicester, UK; 7 Bradford Teaching Hospitals NHS Foundation Trust, Bradford UK; 8 Alexandra Hospital, Cheadle UK; 9 The Leeds Teaching Hospitals NHS Trust Leeds, UK; 10 North-Lincolnshire & Goole Hospitals NHS Trust, Scunthorpe UK; 11 Stockport NHS Foundation Trust, UK; 12 Portsmouth Hospitals University Trust, Portsmouth UK; 13 Oncology Institute of Southern Switzerland, Bellinzona and University of Southern Switzerland, Lugano, Switzerland; 14 Frimley Health NHS Foundation Trust, Slough UK; 15 Torbay and South Devon NHS Foundation Trust, Torbay, UK; 16 Raigmore Hospital, Inverness, UK; 17 Darent Valley Hospital, Dartford, UK; 18 Faculty of Education Health and Wellbeing, University of Wolverhampton, Walsall, UK; 19 Singleton Hospital, Swansea University Health board, Swansea UK; 20 University Hospital of Wales, Cardiff, Heath Park, Cardiff UK; 21 Royal Marsden NHS Foundation Trust and Institute of Cancer Research, London, UK; 22 Lincoln County Hospital, Lincoln, UK; 23 The Hillingdon Hospitals NHS Foundation Trust and Imperial College Healthcare NHS Trust, London, UK; 24 Sussex Cancer Centre, University Sussex Hospitals Trust, Brighton UK; 25 Forth Valley Royal Hospital, Larbert, UK and Beatson West of Scotland Cancer Centre, Glasgow, UK; 26 Ipswich Hospital, East Suffolk and North Essex Foundation Trust Suffolk Ipswich, UK; 27 Division of Cancer and Genetics, Cardiff University, Cardiff, UK; 28 Cancer Institute, University College London, London, UK and University College London Hospitals, London, UK; 29 Institute of Cancer Research, Sutton, UK; 30 The Christie and Salford Royal Hospitals, Manchester UK and University of Manchester Division of Cancer Science UK

## Abstract

**Background:**

Transdermal estradiol (tE2) is an alternative to Luteinizing Hormone Releasing Hormone agonists (LHRHa) for androgen deprivation therapy in prostate cancer management. tE2 suppresses testosterone, mitigates the estrogen-depletion side-effects of LHRHa and the thromboembolic effects of oral estrogen.

**Methods:**

We conducted a randomized, non-inferiority phase III evaluation in locally advanced (M0 N0/N+) prostate cancer comparing LHRHa with tE2 patches (releasing 100 mcg/24h). The primary outcome measure was metastasis-free survival (time from randomization to metastases or death from any cause). The non-inferiority margin was a 4% absolute detriment in 3-year metastasis-free survival (predicted to correspond to a hazard ratio (HR) of 1.27, 85% power, 5% 1-sided significance.) Secondary outcomes included castration rates, toxicity, and overall survival.

**Results:**

Between 2007-2022, 1360 patients were recruited from 75 UK centres, median age 72 years (IQR 68-77), 85% T3, 65% N0. 3-year metastasis-free survival in the LHRHa arm was 86% and 87% for tE2 HR 0.96 (upper-end of the 95% 1-sided CI was 1.11, below the non-inferiority margin of 1.31 derived from the observed metastasis-free survival). Sustained castration rates (testosterone <1.7nmol/L (50ng/dL), over 1 year), with tE2 patch use confirmed as estradiol >250 pmol/L, were 85% in both arms. 5-year overall survival was 79% in LHRHa arm and 81% in tE2 arm (HR 0.90 (95% CI 0.75-1.07)). Adverse events while receiving allocated treatment: any grade hot flushes LHRHa 89%, tE2 44% (grade ≥ 2: 37% versus 8%), any grade gynecomastia LHRHa 42%, te2 85% (grade ≥ 2: 9% versus 37%).

**Conclusion:**

Androgen deprivation therapy using tE2 patches was non-inferior to LHRHa for the endpoint of metastasis-free survival in locally advanced prostate cancer and was associated with reduced hot flushes but greater incidence of gynecomastia.

(Funded by Cancer Research UK and other; **Trial Registration Numbers:** PATCH NCT00303784 and STAMPEDE-1 NCT00268476)

## Introduction

Globally, prostate cancer is the second most common male cancer (1). Reducing serum testosterone to castrate levels is the backbone of therapy for locally advanced non-metastatic (M0) and metastatic (M1) disease (2). This is currently most commonly achieved with Luteinizing Hormone Releasing Hormone agonists (LHRHa) which also reduce serum estradiol as approximately 80% of estrogens in men are derived from the aromatization of testosterone (3). Consequently, LHRHa cause several toxicities including erectile dysfunction and loss of muscle mass attributed to testosterone reduction, as well as loss of bone mineral density increasing the risk of osteoporosis and fracture, adverse metabolic changes (increased serum lipids, glucose and blood pressure), and hot flashes all attributed to a reduction in estradiol (4–6).

An alternative approach is the administration of exogenous estrogen which, through a negative feedback loop on the hypothalamus and pituitary, lowers testosterone (7) but mitigates the estrogen-depletion effects. This approach was first investigated using an oral estrogen (stilbestrol) which lowers testosterone and improves prostate cancer outcomes but was associated with an increase in thromboembolic events (8) attributed to first-pass hepatic metabolism and an increase in liver-derived plasma proteins and coagulation factors (9). Administering estrogen parenterally using a transdermal patch, avoids first-pass hepatic metabolism and therefore lowers the risk of cardiovascular thromboembolic complications associated with oral estrogen (10).

We tested this approach within an adaptive phase II/III transdermal estradiol (tE2) evaluation program for prostate cancer (Figure S1). We initially performed a randomized phase II assessment (n=200) comparing LHRHa versus tE2 patches with a primary outcome measure of cardiovascular morbidity and mortality and demonstrated improved metabolic parameters and no early evidence of increased cardiovascular risk (11). Further data confirmed the preservation of bone mineral density and improved quality of life (QoL) scores with tE2 compared to LHRHa (12, 13). Recruitment was then extended and within a cohort of 1694 men, we confirmed no excess cardiovascular toxicity with longer term follow-up (10). We then expanded the program into 2 separate phase III trials for patients with either M0 or M1 prostate cancer comparing LHRHa to tE2 patches focussing on prostate cancer efficacy. Here we report the results of the M0 evaluation.

## Methods

### Study Design and Trial Oversight

The tE2 prostate program is an academically led, seamless, adaptive phase II/III randomized evaluation (14). The study was initially sponsored by Imperial College London and, from 2020, by University College London (UCL) and conducted by the UK Medical Research Council (MRC) Clinical Trials Unit at UCL. Patients were recruited from the PATCH trial (NCT00303784) network of sites and from 2017 also through the STAMPEDE-1 trial (NCT00268476) network. STAMPEDE-1 is an established adaptive multi-arm multi-stage trial platform that permits the addition of new trial questions (15). UK MRC provided funding throughout the study. Cancer Research UK approved the trial design and provided funding for staff to gather and analyze the data. The trial was conducted in accordance with the principles of Good Clinical Practice guidelines and the Declaration of Helsinki. Appropriate regulatory and ethics approvals were obtained.

### Patients

Patients with histologically confirmed, localized adenocarcinoma of the prostate due to start androgen deprivation therapy (ADT), were eligible. Staging required either T3/4, N0 or NX, M0, assessed with contemporary imaging (initially bone scan and CXR and subsequently CT and MRI) with a prostate specific antigen (PSA) ≥ 20 ng/ml or Gleason sum ≥6, or Tany, N+, M0. Patients relapsing following radical local radiotherapy were required to have a PSA ≥ 4ng/ml and doubling within 6 months, or PSA ≥20ng/ml or be N+. Patients with a significant history of cardiovascular disease as previously described (10) or unfit for protocol therapies were excluded. Eligibility criteria within the PATCH and STAMPEDE-1 protocols were closely aligned.

### Trial procedures

Men were randomly allocated using a computer-based minimization algorithm with a random element (20%) to either LHRHa according to local practice or tE2 patches without blinding. The randomization ratio was 1:1 except for the first 200 patients, where the ratio was 1:2 (LHRHa: tE2) to increase early experience with the patches. Stratification factors common to both trials included age, disease status, brand of LHRHa and intention to give radical prostate radiotherapy and/or adjuvant docetaxel. Additionally, PATCH stratified patients by smoking status, history of cardiac disease in a first-degree relative, center and PSA at entry; STAMPEDE included WHO performance status (PS) and use of non-steroidal anti-inflammatory drugs as stratification factors.

LHRHa subcutaneous injections were administered 4- or 12-weekly, either bicalutamide or flutamide for < 8 weeks were permitted to prevent a tumor flare. Patches releasing 100mcg estradiol per 24 hours (Progynova® or Femseven®) were self-administered. Initial dose was 4 patches twice weekly. If serum testosterone was <1.7 nmol/l (50 ng/dl) at 4 weeks, dosing was reduced to 3 patches twice weekly. Levels of estradiol, testosterone, and PSA were monitored at 4, 12, and 26 weeks, then 6 monthly. Target levels of estradiol were > 250 and < 2000 pmol/L. Evolving standards of care incorporated into the protocol included radiotherapy to the prostate from January 2014 (16), concomitant docetaxel up to 6 cycles from October 2015 (17) and decreasing the duration of ADT to a minimum of 2 years (18). Prophylactic irradiation of the breast area (8 Gy single fraction) to prevent gynecomastia was permitted. At disease progression patient management was at the physician’s discretion including crossing over to the alternate mode of ADT and adding additional therapies.

### Statistical Design and Methods

The primary outcome measure was metastasis-free survival defined as the time from randomization to confirmed metastases (excluding pelvic lymph node progression) or death from any cause. Metastasis-free survival is a recognized surrogate for overall survival (19). To rule out a 4 percentage point absolute detriment in 3-year metastasis-free survival from 83% in the LHRHa arm to 79% in the tE2 arm, corresponding to a hazard ratio (HR) of 1.27 with 85% power and a one-sided 5% significance level, it was estimated that 510 events would be required. Secondary outcome measures included castration rates, toxicities, and overall survival.

Data for this analysis were frozen in the database on 27^th^ February 2025 with pre-specified criteria for being considered as having up-to-date follow-up of confirmed date of death, confirmed trial withdrawn/lost to follow-up, or alive with recent follow-up after January 2023. The primary outcome measure was analyzed employing a meta-analysis type approach with a fixed effects model. Patients recruited through PATCH sites (allocation ratio 2:1), PATCH sites (allocation ratio 1:1) and STAMPEDE-1 sites were treated as 3 separate cohorts. Cox models were used to generate HRs, including terms for age (<70, ≥70), baseline PSA (<50, 50 - <500, ≥500), N-stage, WHO PS status, prior use of LHRHa, intention to give radiotherapy, and intention to give docetaxel. The target HR to demonstrate non-inferiority was based on the observed 3-year metastasis-free survival probability in the LHRHa arm of 86% (based on data pooled across the 3 cohorts). To maintain the absolute pre-specified 4 percentage point non-inferiority margin, under proportional hazards and assuming an exponential survival distribution, a reduction from 86% to 82% in 3-year metastasis-free survival corresponds to a hazard ratio of 1.31. All analyses were performed as intention-to-treat unless specified otherwise. No adjustments for multiple testing were applied.

Patients from the PATCH trial were included in the castration rate analyses if they were continuing the allocated treatment and had a testosterone measurement within the following parameters: +/-1-week around the 4-week time point post-randomisation and +/-4-weeks around later time points. STAMPEDE-1 patients did not have testosterone recorded at baseline nor subsequently on the control arm and are therefore not included in these analyses. Testosterone levels were also analyzed as a time-to-event as previously described (20) with any result within 35 days of randomization excluded to account for the initial testosterone surge seen with LHRHa. Patients who stopped treatment before a non-castrate result (testosterone > 1.7 nmol/l) and those with a serum estradiol value < 250 pmol/L were censored for this castration analysis. For the toxicity analyses, at least one follow-up form with toxicity data had to have been received (n=1348) and data were recorded using common terminology criteria for adverse events (CTCAE). Fracture rate data were obtained from PATCH toxicity and serious adverse event forms and analyzed using competing risk survival methods with death as a competing risk.

## Results

**Figure 1:**
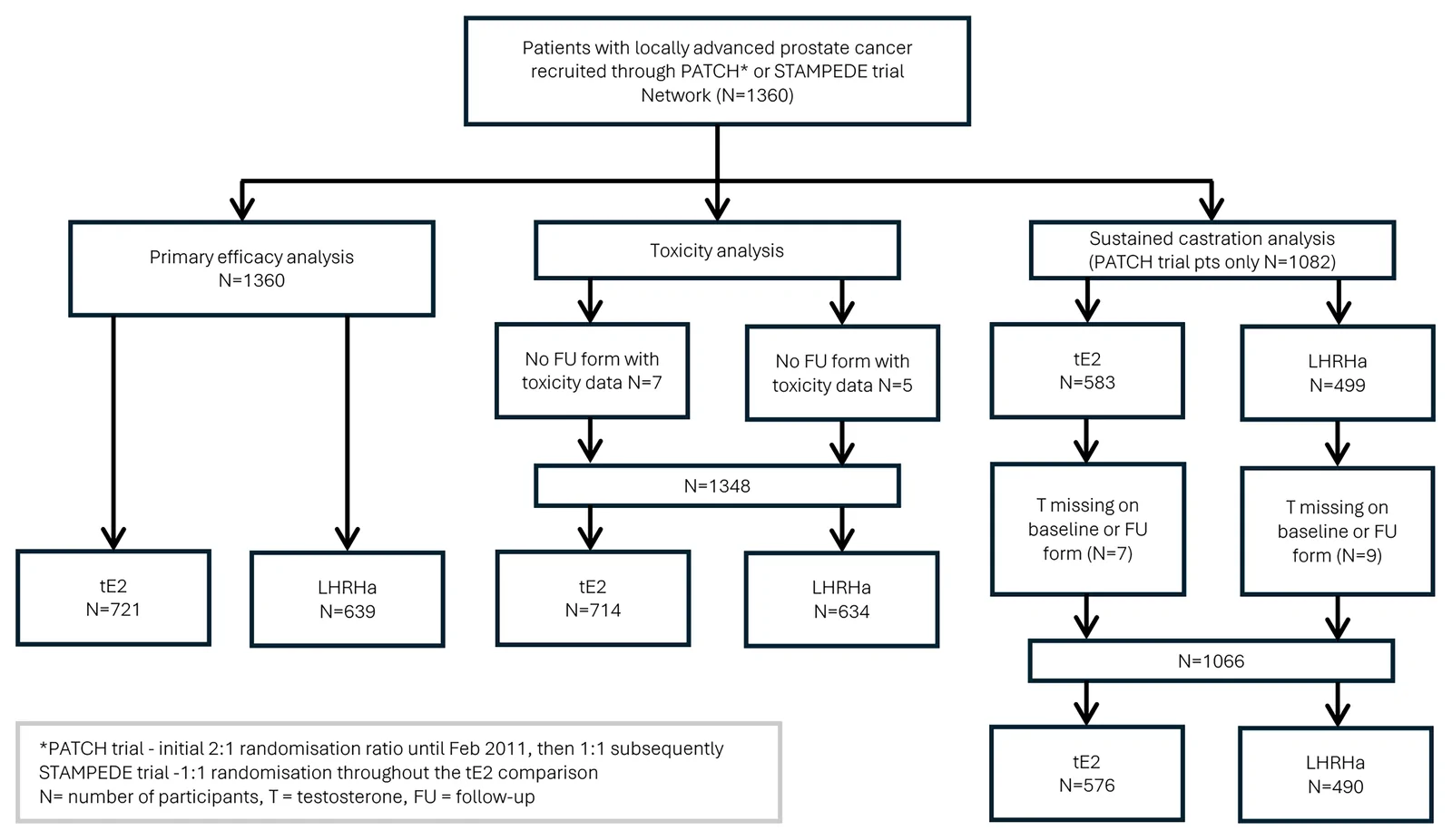
Consort Diagram

Between 2007 and 2022, 1360 patients were recruited (1,082/1,360 (80%) from PATCH sites and from 2017, 278/1,360 (20%) from STAMPEDE-1 sites. Forty-seven percent (639/1,360) were allocated to LHRHa and 53% (721/1,360) to tE2, reflecting the shift from the initial allocation ratio of 1:2 to 1:1 (Consort diagram Figure 1). Only 35/1,360 (<3%) did not meet the pre-specified criteria defined above as having up-to-date follow-up. Characteristics at baseline were balanced between randomized groups (Table 1). The median age was 72 years (interquartile range (IQR)) 68-77), with 66% (897/1,360) ≥ 70 years and 76% (1,032/1,360) having a WHO PS status of 0 (a 5-point scale where higher numbers reflect greater disability and 0 is asymptomatic). The median PSA was 24.4 ng/ml (IQR 11.8- 54.1) at trial entry; 60% (811/1,360) had a Gleason score of 8-10, 85% (1,157/1,360) were T3, and 65% (883/1,360) were node negative. Radiotherapy to the prostate was planned in 68% (928/1,360) (this change in standard care was introduced during trial recruitment) and the use of concomitant docetaxel also introduced during recruitment was planned in 6% (80/1,360). Patients recruited through the two trial networks, and between the PATCH 2:1 and 1:1 cohorts, were similar (Table S1). Patients within the 2:1 cohort (11% (144/1,360) of the total cohort) were older, with 110/144 (76%) ≥70 years, and 91/144 (63%) were PS 0. In contrast, STAMPEDE-1 patients were younger, with 55% (152/278) ≥ 70 years with 87% (242/278) having a PS of 0, 79% (220/278) with a Gleason score of 8-10, and 92% (257/1,365) planned for prostate radiotherapy, reflecting the more recent recruitment of this group.

At the time of analysis, 568 metastasis-free survival events had occurred, the 3-year LHRHa metastasis-free survival was 85.6% and was 87.1% for tE2 (difference +1.2%, 95% CI for difference -2.5% to +4.9%); HR 0.96 (95% 2 sided CI 0.81-1.14) in favor of tE2 with an upper-end of a 95% 2-sided CI of 1.14 (Figure 2A). The non-inferiority margin was 1.31 thus non-inferiority has been demonstrated, equivalent to excluding a 2 percentage point absolute different in metastasis-free survival. Metastasis-free survival by the trial networks, PATCH trial allocation ratio effect of radiotherapy and 2-sided 95% CI is shown in Figure S2A and B. 5-year overall survival was 79.2% in LHRHa arm and 81.1% in tE2 arm difference +1.8% 95% CI of difference -2.6% to +6.2% (HR was 0.90 (95% CI 0.75-1.07)) (Figure 2B), with overall survival by trial network and PATCH trial allocation ratio illustrated in Figure S2C. Causes of death are detailed in Table S2. In total, 501/1360 (37%) patients had died, 213/501 (43%) from prostate cancer. Other significant causes of death included second primary malignancies 65/501 (13%), cardiovascular causes 60/501 (12%), and respiratory causes including bronchopneumonia 79/501 (16%). Causes of death between randomized groups were similar.

1075/1082 patients from the PATCH network contributed to the data in Figure 3A, which shows castrate testosterone levels (<1.7 nmol/l (50 ng/dL)) over the first year. One-month castration rates were better for tE2 than LHRHa due to the absence of a flare reaction, with similar levels maintained over subsequent months. 1066/1082 patients contributed to the sustained castration rate analysis over the first year. For 16 patients (9 LHRHa, 7 tE2) testosterone levels were missing either on the baseline or follow-up forms and they were excluded. Figure 3B shows similar sustained castration rates for patients remaining on allocated treatment, with tE2 adherence defined as having a serum estradiol >250 pmol/L, and for LHRHa, having a confirmed date of last injection. Castration rate analyses by target testosterone level and brand of patch are shown in Table S4.

Estradiol levels over time during the first-year post-randomization are shown in Table S3, where the mean levels for patients on tE2 remained fairly constant around 900 pmol/L. Those randomized to receive tE2 remained on allocated treatment for a median of 3.25 (95% CI 3.05 –3.44) years versus 4.27 (95% CI 3.95 – 4.58) years for LHRHa. This was accounted for by tE2 patients switching to LHRHa (but not vice-versa as the patches were still under-evaluation) for either toxicity or on progression.

Adverse events experienced by the end of the allocated treatment are shown in Table 2. The number of patients reporting grade ≥ 3 adverse events was low. Side effects related to low testosterone levels were similar in both arms, with approximately 65% reporting erectile dysfunction and around 60% reporting decreased libido. Differences between treatments were as anticipated, more participants experiencing hot flushes with LHRHa 89% versus 44% for tE2, (grade 2: 32% versus 7%, grade ≥ 3 5% versus <1%)). Gynecomastia developed in 42% with LHRHa versus 85% with tE2 (grade 2: 8% versus 33%, grade 3 1% versus 5%). Prophylactic breast irradiation was given in 46/576 (8%) of PATCH patients allocated to tE2; gynecomastia rates while on allocated treatment were similar in those with irradiation (grade 0-1 58%, grade 2-3 42%) and those without irradiation (grade 0-1 59%, grade 2-3 41%). Rates of pruritus were higher with the patches, while rates of insomnia and fatigue were lower. The 5- and 10-year fracture rate for PATCH patients was 5.8% and 10.5% for LHRHa versus 2.8% and 5.1% tE2.

## Discussion

This large, randomized trial data set shows that tE2 patches were noninferior to LHRHa for metastases-free survival in men with high-risk non-metastatic prostate cancer. 5-year overall survival was 79% in the LHRHa arm and 81% in the tE2 arm (HR was 0.90 (95% CI 0.75-1.07)). The benefits of tE2 include reduced loss of bone mineral density, decreased fracture rates, better metabolic profiles, and fewer hot flushes (10, 12, 13), albeit with a higher rate of gynecomastia. These data give patients choice regarding expected side effects and the route of administration, information that should be incorporated into discussions with patients before starting ADT. The need for more information and educational programs about ADT for patients and their partners has been highlighted (4, 21).

The effectiveness of reducing serum testosterone to control prostate cancer growth was first recognized by Huggins and Hodges (22). It was initially achieved with surgical castration or oral estrogen (8) until the advent of LHRHa in the 1980s (23). Current pharmaceutical options for ADT include injectable depot LHRH agonists, injectable or oral antagonists (20, 24) and now tE2 patches. To inform this decision several issues should be considered. First, the views of the patient are important. Data from focus groups suggest that patients will have a preferred approach to ADT for both practical (particularly avoiding injections) and emotional reasons (25).

Reductions in serum estradiol that accompany both injectable and oral LHRH analogues cause many of the side effects associated with ADT (Figure S3) (6). Hot flushes burden many prostate cancer patients significantly affecting their QoL (26). In a randomized cohort of 737 men, mean QoL scores were higher for those receiving tE2 versus LHRHa as ADT, attributed to fewer hot flushes, reduced fatigue and improved physical function (13). Forty-six percent receiving LHRHa versus 8% receiving tE2 were “quite a bit” or “very” bothered by hot flushes. Rates of patient-reported gynecomastia were 5% for LHRHa compared to 37% for tE2, consistent with the data reported here.

A second consideration is whether the mode of ADT is compatible with other currently recommended treatments. Throughout the evaluation of tE2, standard of care has evolved with prostate radiotherapy and docetaxel now given concomitantly with conventional ADT (16, 17). These advances were incorporated into our studies with enhanced monitoring for tE2 patients to ensure no excess toxicity resulted from using the patches with either new treatment. Most recently, androgen receptor pathway inhibitors have been introduced (27). Combining these with tE2 has been assessed within the M1 PATCH trial. PSA response in the first 6 months was similar in patients receiving androgen receptor pathway inhibitors with either tE2 or LHRHa (28).

A third consideration is longer-term health outcomes following a prostate cancer diagnosis and management. Bone mineral density loss and subsequent osteoporosis from current ADT is associated with significant morbidity and mortality (29). Comparing bone mineral density in 74 prostate cancer patients using dual-energy x-ray absorptiometry scans 1 year after commencing either LHRHa or tE2 patches, mean percentage change was -1.4% for men receiving LHRHa compared to +6.0% for those receiving tE2 (p<0.001) (12). Strategies to mitigate bone mineral density loss during ADT include the addition of bone strengthening agents such as bisphosphonates (30). However, this increases polypharmacy and treatment costs, with real-world data indicating that ADT bone toxicity is poorly managed (31). With prostate cancer incidence increasing in low-and-middle-income countries (32), a self-administered, low-toxicity lower cost therapy that mitigates the need for bone protection agents is an attractive option, with these benefits also applying in high-income settings. Example comparative costs are given in the Supplementary Appendix.

Strengths of our study include the adaptive trial design and an extended follow-up period, allowing for long-term monitoring of toxicities and confirmation of our original hypothesis that parenteral administration will deliver the benefits of estrogen on prostate cancer outcomes while avoiding the cardiovascular thromboembolic side effects (10). Limitations include that prostate radiotherapy was introduced during the study, and the duration of ADT and systemic treatments evolved during the trial (33). Time on allocated treatment was shorter for tE2 patches compared to LHRHa patients accounted for by patients switching to LHRHa from tE2 for toxicity or progression but very rarely vice-versa as the patches were still under evaluation. This does however demonstrate that patients can transition from one mode of ADT to another if they experience significant side effects. Only 8% of patients received prophylactic breast irradiation which did not appear to ameliorate the gynecomastia. Comparative data on testosterone recovery is not available. Data on quality of life from validated questionnaires were collected and have not yet been analyzed. Finally, we acknowledge that other forms of tE2 exist such as gels that could be used for ADT (34).

In summary, tE2 patches reduce testosterone to castrate levels effectively. Compared to LHRHa, they have non-inferior effects on metastasis-free survival. Allocation to tE2 reduced hot flushes which can be debilitating, and fracture rates were lower; gynecomastia rates were increased. tE2 patches can therefore be considered an alternative choice for testosterone suppression in men with M0/N0/N+, prostate cancer. The patches appear equally effective as the standard LHRHa approach against prostate cancer and are associated with a lower incidence of both the short and long-term estrogen-depletion deleterious side effects of LHRHa treatment.

Disclosure forms provided by the authors are available with the full text of this article at NEJM.org.

## Supplementary Material

Supplement

## Figures and Tables

**Figure:**
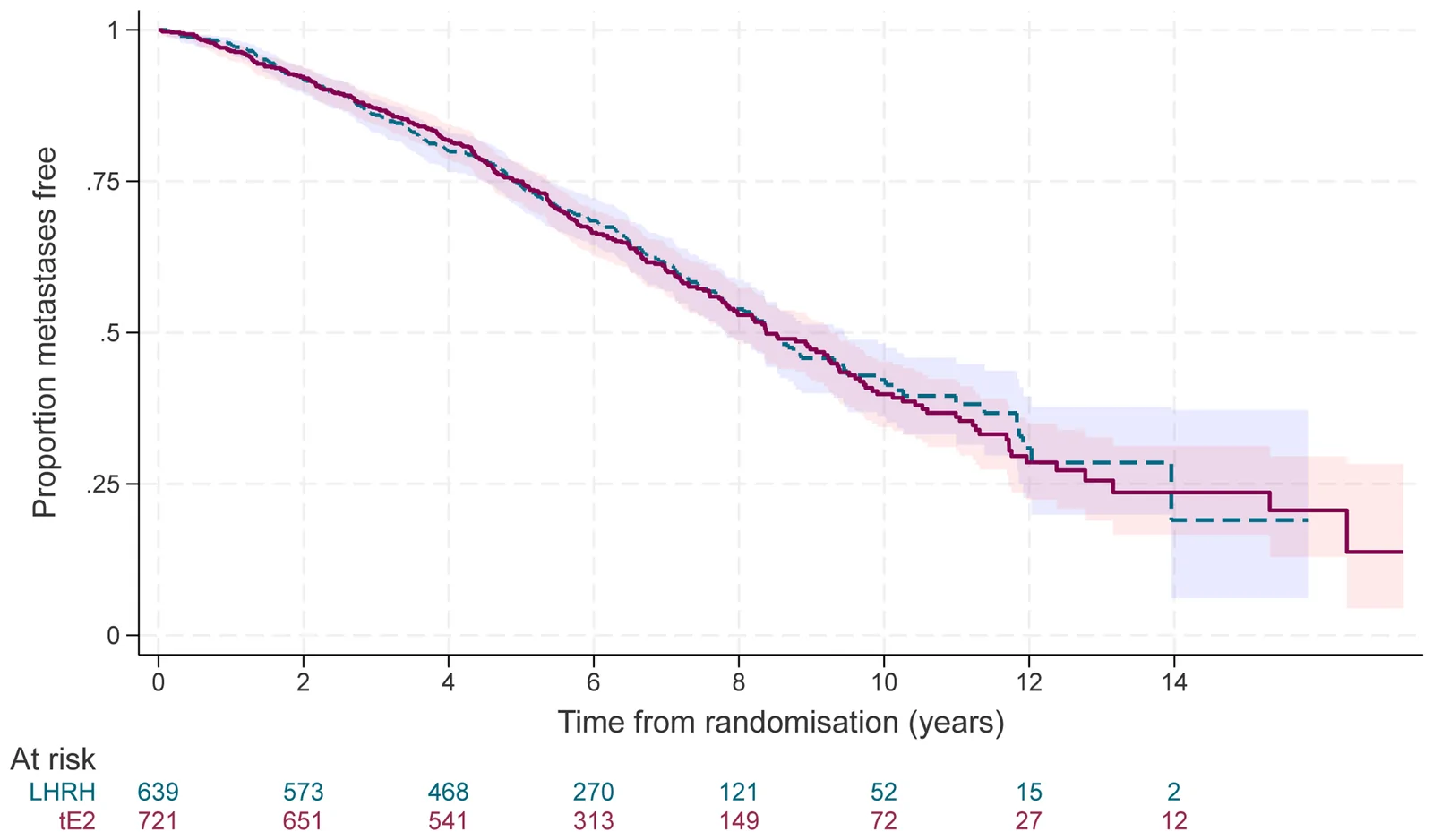


**Figure:**
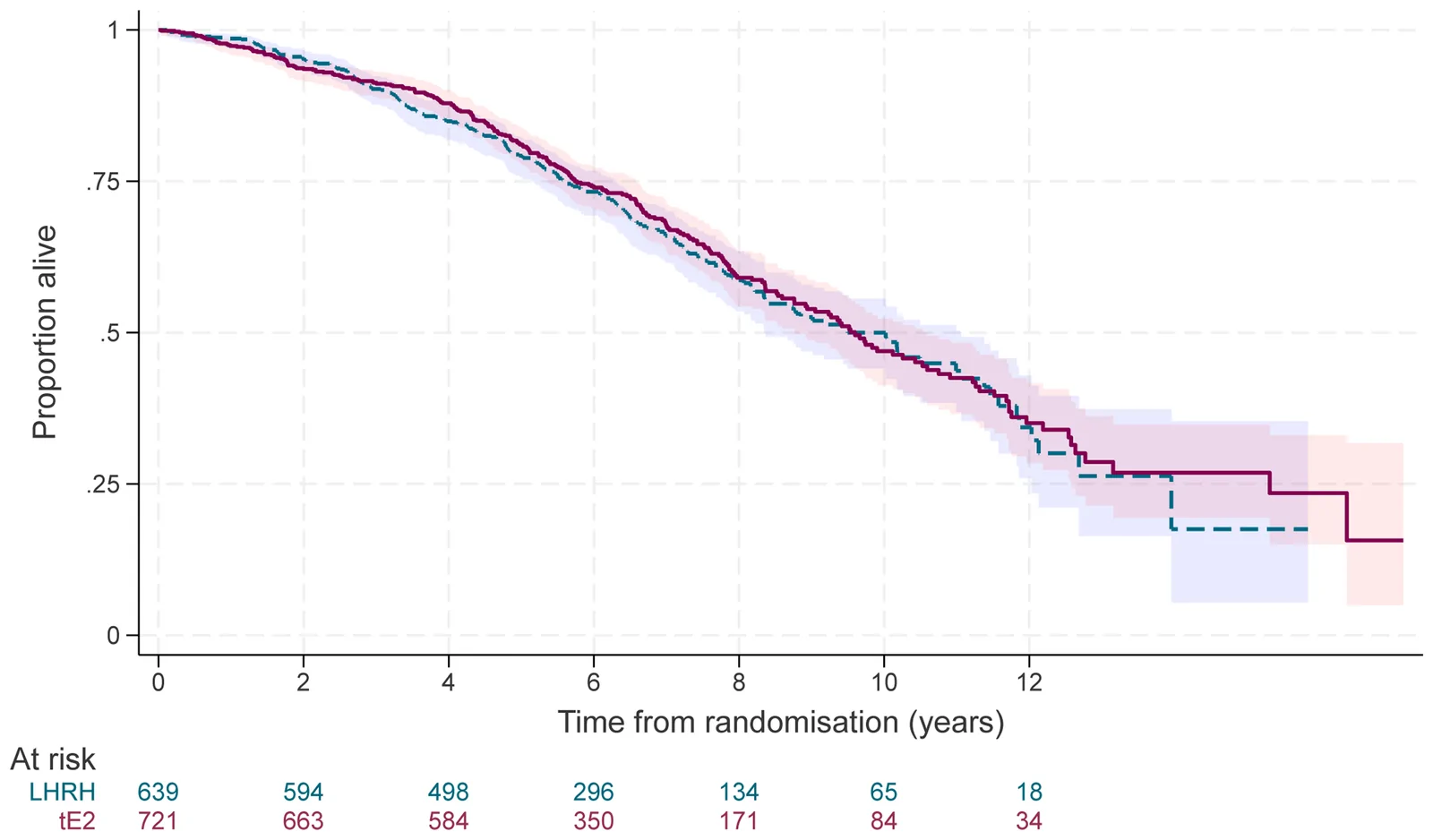


**Figure:**
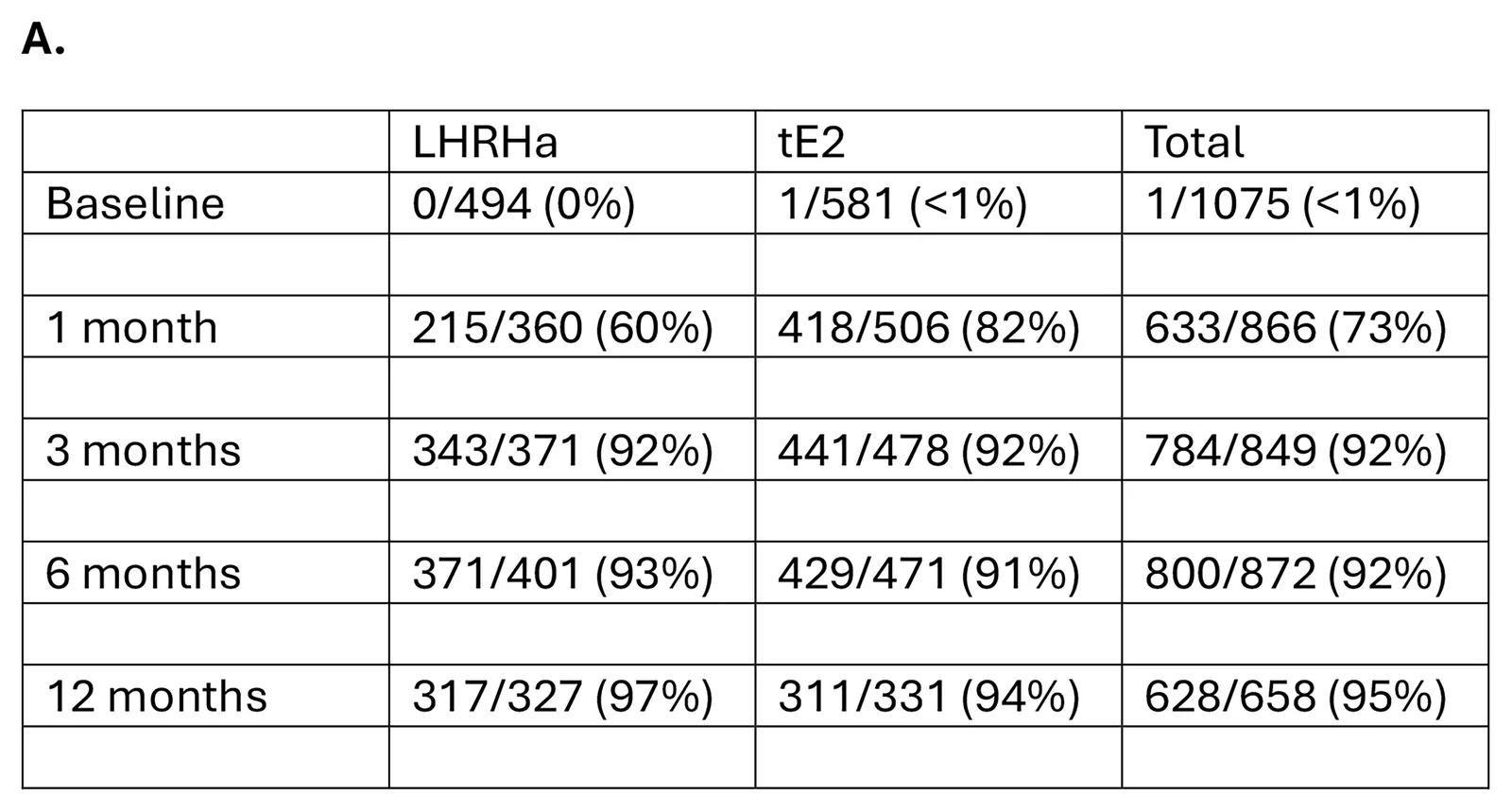


**Figure:**
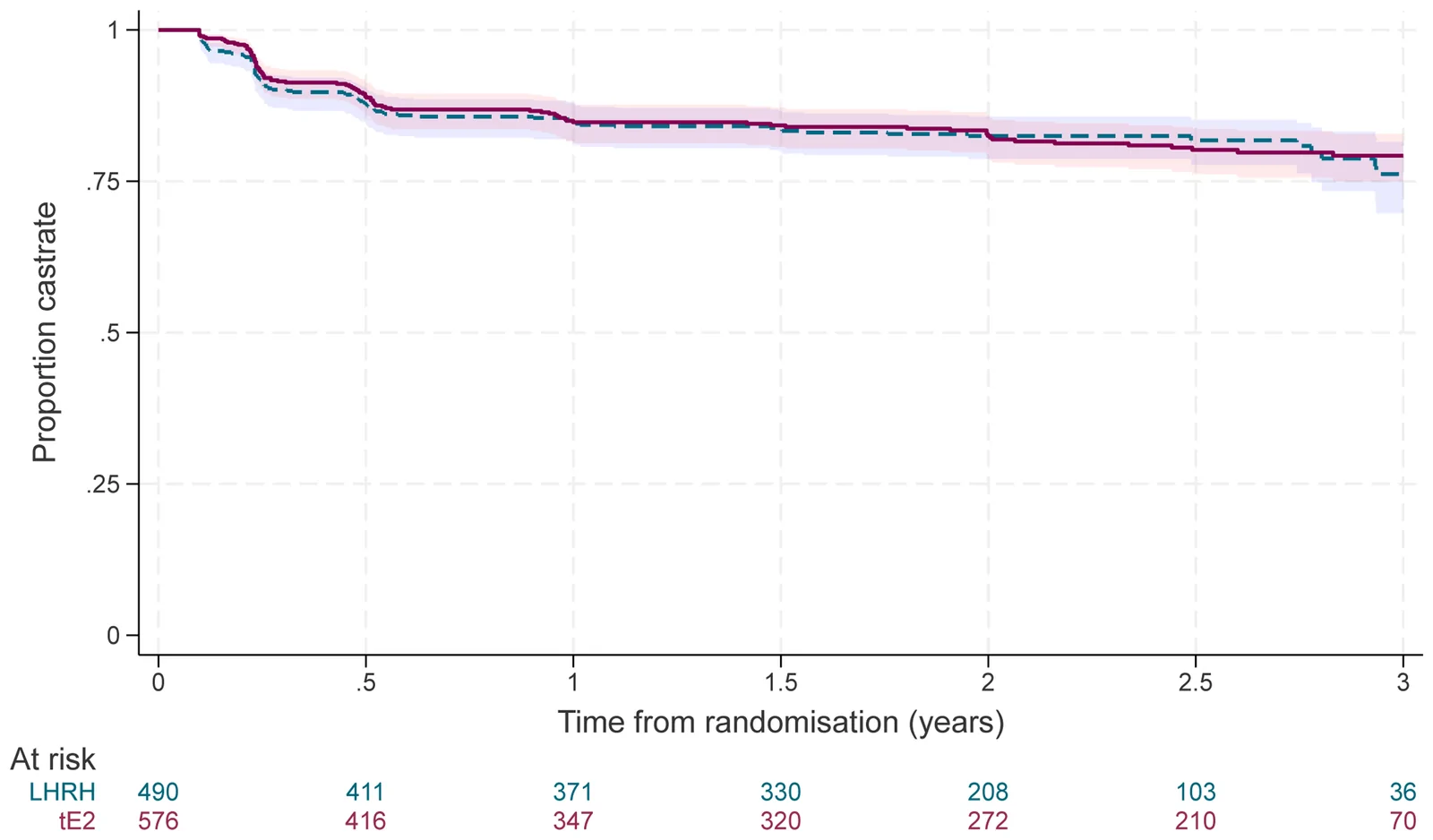


**Table 1:** Baseline Characteristics

	Treatment Arm					
	LHRHa N=639		tE2 N=721		Total N=1360	
	No.	%	No.	%	No.	%
**Trial**						
PATCH	499	78%	583	81%	1,082	80%
STAMPEDE	140	22%	138	19%	278	20%
**Age**						
Median (IQR)	72 (67 – 77)		72 (68 – 77)		72 (68 – 77)	
Range	50 – 89		46 – 90		46 – 90	
<70	219	34%	244	34%	463	34%
>=70	420	66%	477	66%	897	66%
**WHO performance status**						
0: Normal activity without restriction	488	76%	544	75%	1,032	76%
1: Strenuous activity restricted, can do light work	139	22%	154	21%	293	22%
2: Up and about >50% of waking hours, limited self-care	12	2%	23	3%	35	3%
**Tumor status**						
T0	1	0%	0	0%	1	0%
T1	5	1%	5	1%	10	1%
T2	21	3%	25	3%	46	3%
T3	542	85%	615	85%	1,157	85%
T4	67	11%	72	10%	139	10%
Tx	2	0%	4	1%	6	0%
**Nodal status**						
N0	427	67%	456	63%	883	65%
N+	150	23%	164	23%	314	23%
NX	62	10%	101	14%	163	12%
**PSA**						
Median (IQR)	23.8 (11.3 – 53.1)		25.21 (12 – 54.9)		24.4 (11.8 – 54.1)	
Range	1.1 – 2488		0.8 – 544.1		0.8 – 2488	
<50	474	74%	519	72%	993	73%
50 – 499.9	161	25%	201	28%	362	27%
>=500	4	1%	1	0%	5	0%
**Gleason Sum Score**						
4	1	0%	0	0%	1	0%
5	5	1%	3	1%	8	1%
6	33	5%	35	5%	68	5%
7	213	33%	253	35%	466	34%
8	139	22%	158	22%	297	22%
9	238	37%	252	35%	490	36%
10	10	2%	13	2%	23	2%
**Gleason Sum Score (grouped)**						
4-6	39	6%	39	5%	77	6%
7	213	33%	253	35%	466	34%
8-10	387	61%	423	59%	810	60%
**RT to prostate planned as part of treatment?**						
No	191	30%	241	33%	432	32%
Yes	448	70%	480	67%	928	68%
**SOC docetaxel / abiraterone planned?**						
No	596	93%	683	95%	1,279	94%
Docetaxel	43	7%	37	5%	80	6%
Abiraterone	0	0%	1	0%	1	0%

**Legend**: Baseline characteristics by allocated treatment. Abbreviations RT = radiotherapy, SOC = standard of care

**Table 2:** Maximum grade of toxicity whilst receiving allocated treatment

	LHRHa				tE2			
	n(%)	n(%)	n(%)	n(%)	n(%)	n(%)	n(%)	n(%)
	None	Grade 1	Grade 2	Grade 3-4	None	Grade 1	Grade 2	Grade 3-4
Gynecomastia	357 (58%)	199 (33%)	47 (8%)	9 (1%)	105 (15%)	329 (47%)	226 (33%)	33 (5%)
Hot flushes	70 (11%)	316 (52%)	195 (32%)	31 (5%)	387 (56%)	252 (36%)	51 (7%)	3 (<1%)
Anaemia	401 (66%)	182 (30%)	25 (4%)	4 (1%)	518 (75%)	161 (23%)	13 (2%)	1 (<1%)
Anxiety	424 (69%)	154 (25%)	32 (5%)	2 (<1%)	519 (75%)	154 (22%)	20 (3%)	0 (0%)
Chest pain	550 (90%)	45 (7%)	10 (2%)	7 (1%)	646 (93%)	39 (6%)	5 (1%)	3 (<1%)
Concentration impairment	462 (75%)	128 (21%)	18 (3%)	4 (1%)	559 (81%)	120 (17%)	12 (2%)	2 (<1%)
Depression	442 (72%)	137 (22%)	30 (5%)	3 (<1%)	537 (77%)	136 (20%)	18 (3%)	2 (<1%)
Dizziness	474 (77%)	124 (20%)	13 (2%)	1 (<1%)	582 (84%)	101 (15%)	9 (1%)	1 (<1%)
Erectile dysfunction	220 (36%)	215 (35%)	145 (24%)	32 (5%)	244 (35%)	240 (35%)	184 (27%)	25 (4%)
Fatigue	126 (21%)	330 (54%)	147 (24%)	9 (1%)	225 (32%)	355 (51%)	106 (15%)	7 (1%)
Insomnia	353 (58%)	204 (33%)	52 (8%)	3 (<1%)	483 (70%)	186 (27%)	20 (3%)	4 (1%)
Increased irritability	439 (72%)	153 (25%)	17 (3%)	3 (<1%)	525 (76%)	155 (22%)	13 (2%)	0 (0%)
Decreased libido	253 (41%)	222 (36%)	125 (20%)	12 (2%)	293 (42%)	238 (34%)	154 (22%)	8 (1%)
Nausea	528 (86%)	76 (12%)	7 (1%)	1 (<1%)	624 (90%)	64 (9%)	4 (1%)	1 (<1%)
Pruritus	493 (81%)	106 (17%)	13 (2%)	0 (0%)	384 (55%)	268 (39%)	36 (5%)	5 (1%)

**Legend**: Toxicity by random allocation and common terminology criteria for adverse events (CTCAE) grading. Patients recruited through the PATCH protocol were assessed using CTCAE version 3 and version 4 for patients recruited through the STAMPEDE protocol. Percentages are presented to the nearest whole percent, and therefore may not sum to exactly 100.

**Table:** 

	LHRHa	tE2	Total
Baseline	0/494 (0%)	1/581 (<1%)	1/1075 (<1%)
			
1 month	215/360 (60%)	418/506 (82%)	633/866 (73%)
			
3 months	343/371 (92%)	441/478 (92%)	784/849 (92%)
			
6 months	371/401 (93%)	429/471 (91%)	800/872 (92%)
			
12 months	317/327 (97%)	311/331 (94%)	628/658 (95%)
			

## References

[1] Bray F, Laversanne M, Sung H, Ferlay J, Siegel RL, Soerjomataram I, Jemal A (2024). Global cancer statistics 2022: GLOBOCAN estimates of incidence and mortality worldwide for 36 cancers in 185 countries. CA Cancer J Clin.

[2] Schaeffer EM, Srinivas S, Adra N, An Y, Barocas D, Bitting R (2023). Prostate Cancer, Version 4.2023, NCCN Clinical Practice Guidelines in Oncology. J Natl Compr Canc Netw.

[3] Finkelstein JS, Lee H, Burnett-Bowie SA, Pallais JC, Yu EW, Borges LF (2013). Gonadal steroids and body composition, strength, and sexual function in men. N Engl J Med.

[4] Walker LM, Tran S, Robinson JW (2013). Luteinizing hormone--releasing hormone agonists: a quick reference for prevalence rates of potential adverse effects. Clin Genitourin Cancer.

[5] Mousa A, Nguyen DD, Lalani AK, Satkunasivam R, Aminoltejari K, Hird A (2024). Metabolic, cardiac, and bone health testing in patients with prostate cancer on androgen-deprivation therapy: A population-based assessment of adherence to therapeutic monitoring guidelines. Cancer.

[6] Coelingh Bennink HJT, Prowse A, Egberts JFM, Debruyne FMJ, Huhtaniemi IT, Tombal B (2024). The Loss of Estradiol by Androgen Deprivation in Prostate Cancer Patients Shows the Importance of Estrogens in Males. J Endocr Soc.

[7] Hayes FJ, Seminara SB, Decruz S, Boepple PA, Crowley WF (2000). Aromatase inhibition in the human male reveals a hypothalamic site of estrogen feedback. J Clin Endocrinol Metab.

[8] Byar DP (1972). Treatment of prostatic cancer: studies by the Veterans Administration cooperative urological research group. Bull N Y Acad Med.

[9] von Schoultz B, Carlstrom K, Collste L, Eriksson A, Henriksson P, Pousette A, Stege R (1989). Estrogen therapy and liver function--metabolic effects of oral and parenteral administration. Prostate.

[10] Langley RE, Gilbert DC, Duong T, Clarke NW, Nankivell M, Rosen SD (2021). Transdermal oestradiol for androgen suppression in prostate cancer: long-term cardiovascular outcomes from the randomised Prostate Adenocarcinoma Transcutaneous Hormone (PATCH) trial programme. Lancet.

[11] Langley RE, Cafferty FH, Alhasso AA, Rosen SD, Sundaram SK, Freeman SC (2013). Cardiovascular outcomes in patients with locally advanced and metastatic prostate cancer treated with luteinising-hormone-releasing-hormone agonists or transdermal oestrogen: the randomised, phase 2 MRC PATCH trial (PR09). Lancet Oncol.

[12] Langley RE, Kynaston HG, Alhasso AA, Duong T, Paez EM, Jovic G (2016). A Randomised Comparison Evaluating Changes in Bone Mineral Density in Advanced Prostate Cancer: Luteinising Hormone-releasing Hormone Agonists Versus Transdermal Oestradiol. Eur Urol.

[13] Gilbert DC, Duong T, Kynaston HG, Alhasso AA, Cafferty FH, Rosen SD (2017). Quality-of-life outcomes from the Prostate Adenocarcinoma: TransCutaneous Hormones (PATCH) trial evaluating luteinising hormone-releasing hormone agonists versus transdermal oestradiol for androgen suppression in advanced prostate cancer. BJU Int.

[14] Gilbert DC, Nankivell M, Rush H, Clarke NW, Mangar S, Al-Hasso A (2024). A Repurposing Programme Evaluating Transdermal Oestradiol Patches for the Treatment of Prostate Cancer Within the PATCH and STAMPEDE Trials: Current Results and Adapting Trial Design. Clin Oncol (R Coll Radiol).

[15] Sydes MR, Parmar MK, Mason MD, Clarke NW, Amos C, Anderson J (2012). Flexible trial design in practice - stopping arms for lack-of-benefit and adding research arms mid-trial in STAMPEDE: a multi-arm multi-stage randomized controlled trial. Trials.

[16] Mason MD, Parulekar WR, Sydes MR, Brundage M, Kirkbride P, Gospodarowicz M (2015). Final Report of the Intergroup Randomized Study of Combined Androgen-Deprivation Therapy Plus Radiotherapy Versus Androgen-Deprivation Therapy Alone in Locally Advanced Prostate Cancer. J Clin Oncol.

[17] James ND, Sydes MR, Clarke NW, Mason MD, Dearnaley DP, Spears MR (2016). Addition of docetaxel, zoledronic acid, or both to first-line long-term hormone therapy in prostate cancer (STAMPEDE): survival results from an adaptive, multiarm, multistage, platform randomised controlled trial. Lancet.

[18] Bolla M, de Reijke TM, Van Tienhoven G, Van den Bergh AC, Oddens J, Poortmans PM (2009). Duration of androgen suppression in the treatment of prostate cancer. N Engl J Med.

[19] Xie W, Regan MM, Buyse M, Halabi S, Kantoff PW, Sartor O (2017). Metastasis-Free Survival Is a Strong Surrogate of Overall Survival in Localized Prostate Cancer. J Clin Oncol.

[20] Shore ND, Saad F, Cookson MS, George DJ, Saltzstein DR, Tutrone R (2020). Oral Relugolix for Androgen-Deprivation Therapy in Advanced Prostate Cancer. N Engl J Med.

[21] Walker LM, Sears CS, Wibowo E, Robinson JW, Matthew AG, McLeod DL, Wassersug RJ (2024). A Non-Randomized Comparison of Online and In-Person Formats of the Canadian Androgen Deprivation Therapy Educational Program: Impacts on Side Effects, Bother, and Self-Efficacy. Curr Oncol.

[22] Huggins C, Hodges CV (2002). Studies on prostatic cancer: I. The effect of castration, of estrogen and of androgen injection on serum phosphatases in metastatic carcinoma of the prostate. 1941. J Urol.

[23] Plosker GL, Brogden RN (1994). Leuprorelin. A review of its pharmacology and therapeutic use in prostatic cancer, endometriosis and other sex hormone-related disorders. Drugs.

[24] Zengerling F, Jakob JJ, Schmidt S, Meerpohl JJ, Blumle A, Schmucker C (2021). Degarelix for treating advanced hormone-sensitive prostate cancer. Cochrane Database Syst Rev.

[25] Rush HL, Merrick S, Marshall J, Deighan J, Abdel-Aty H, Alhasso A (2025). Preferences between three options for androgen deprivation therapy: a focus group study. BJU Int.

[26] Nguyen PL, Alibhai SM, Basaria S, D’Amico AV, Kantoff PW, Keating NL (2015). Adverse effects of androgen deprivation therapy and strategies to mitigate them. Eur Urol.

[27] Jazayeri SB, Srivastava A, Shore N (2022). Review of second-generation androgen receptor inhibitor therapies and their role in prostate cancer management. Curr Opin Urol.

[28] James N (2025). TE2 +ARPi. GU ASCO abstract.

[29] Jones C, Gray S, Brown M, Brown J, McCloskey E, Rai BP (2024). Risk of Fractures and Falls in Men with Advanced or Metastatic Prostate Cancer Receiving Androgen Deprivation Therapy and Treated with Novel Androgen Receptor Signalling Inhibitors: A Systematic Review and Meta-analysis of Randomised Controlled Trials. Eur Urol Oncol.

[30] Brown JE, Handforth C, Compston JE, Cross W, Parr N, Selby P (2020). Guidance for the assessment and management of prostate cancer treatment-induced bone loss. A consensus position statement from an expert group. J Bone Oncol.

[31] El Badri AM, Salawu A, Brown JE (2019). Bone Health in Men with Prostate Cancer: Review Article. Curr Osteoporos Rep.

[32] Culp MB, Soerjomataram I, Efstathiou JA, Bray F, Jemal A (2020). Recent Global Patterns in Prostate Cancer Incidence and Mortality Rates. Eur Urol.

[33] Azad AA, Kostos L, Agarwal N, Attard G, Davis ID, Dorff T (2025). Combination Therapies in Locally Advanced and Metastatic Hormone-sensitive Prostate Cancer. Eur Urol.

[34] Watson RWR, Schellhammer P (2025). The case for, and challenges to using transdermal estradiol for androgen deprivation therapy. Journal of Urology.

[35] Janopaul-Naylor J, Yadalam AK, Chan JSK, Dee EC, Sun YV, Cantu SM (2025). Modifiable Cardiovascular Risk Factors Amongst Men With and Without Prostate Cancer in a Large, Prospective Registry. Clin Genitourin Cancer.

