# Supplementary material for: Transdermal Estradiol Patches for ADT in Locally Advanced Prostate Cancer
Source: N Engl J Med. Author manuscript; Available in PMC 2026 Nov 2. (PMC7619680; doi:10.1056/NEJMoa2511781)
Supplement: Supplementary file 1 [file EMS213529-supplement-Supplement.pdf]

Appendix: Transdermal Estradiol for ADT in M0 Prostate Cancer

Table of Contents **Page No**

Index	1
PATCH and STAMPEDE Centres and Lead investigators that Enrolled Patients	2
Supplementary Table 1: Baseline Characteristics by Trial Network and Randomization Ratio	4
Supplementary Table 2: Causes of Death	6
Supplementary Table 3: Estradiol at Different Time Points	7
Supplementary Table 4A: Castration Rates by Target Testosterone Levels	8
Supplementary Table 4B: Castration Rates by Make of Patch Over Time	8
Supplementary Figure 1: Overview of the tE2 Evaluation Program	9
Supplementary Figure 2A: Metastasis Free Survival by Trial Network and PATCH Randomization Ratio with i) 90% and ii) 95% confidence intervals shown	10
Supplementary Figure 2B: Metastasis-Free Survival - Effect of Prostate Radiotherapy	10
Supplementary Figure 2C: Overall Survival by Trial Network and PATCH Randomization Ratio	11
Supplementary Figure 3: Androgen Deprivation Therapy with LHRHa	12
Additional Information i) Further Statistical Detail and ii) Exemplar Costs	13

PATCH and STAMPEDE Centres and Investigators that Enrolled Patients

Addenbrooke's Hospital: Danish Mazhar, Helen Patterson; Airedale Hospital: Simon Brown, Robert Brierly; Ayr Hospital: Hilary Glen, Jawaher Ansari; Barnet Gen Hosp: Sarah Needleman; Bath Royal United Hospital: Jonathan McFarlane; Birmingham, QE: David Fackrell, Nicholas James; Blackburn East Lancs Trust: Omi Parikh; Bolton, Royal Hospital: Ling Lee; Boston, Pilgrim Hospital: Miguel Panades; Bradford Royal Infirmary: Rohit Chahal, Simon Brown; Brighton, Royal Sussex County Hosp: Angus Robinson; Burton, Queen's Hospital: Mike Smith-Howell; Cardiff, Velindre: Jacob Tanguay; Castle Hill Hospital: Matthew Simms, John Hetherington ;Charing Cross Hospital: Stephen Mangar, Paul Abel, Alison Falconer ; Clatterbridge Centre for Oncology: Isabel Syndikus, Zafar Malik ; Crewe, Leighton Hospital: Anna Tran; Darent Valley Hospital: Sanjeev Madaan; Derbyshire Royal Infirmary: Simon Williams, Prantik Das; Diana Princess of Wales, Grimsby: Sanjay Dixit , Iqtedar Muazzam; Eastbourne District Hospital: Duncan Gilbert, Caroline Manetta; Essex County/Colchester General Hospital: Dakshinamoorthy Muthukumar, Bruce Sizer; Exeter, RD&E: Mohini Varughese; Forth Valley Royal Hospital: Stephen McKay, Michael Mehir, Seamus Teahan; Freeman Hospital, Newcastle: Edgar Paez, Garrett Durkan, P Powell, Ashraf Azzabi; Glasgow Beatson Oncology Centre: Abdulla Alhasso, John Graham, Rob Jones; Great Western Hospital: Omar Khan, David Cole; Guildford, Royal Surrey County Hospital: Carla Perna; Halton Hospital: Isabel Syndikus; Harlow, Princess Alexandra Hospital: Lucinda Melcher; Hillingdon Hospital: Alvan Pope; Hope Hospital/Salford Royal: Noel Clarke; Huddersfield Royal Infirmary: Uschi Hofmann, Uschi Hofmann; Inverness, Raigmore Hospital: Robert Dealey; Ipswich Hospital: Ramachandran Venkitaraman, Chris Scrase, Neil McPhail; James Cook Univ Hospital: Clive Peedell, David Chadwick, John Hardman; Kent Oncology Centre: Henry Taylor, Sharon Beesley; Kidderminster: Mark Churn; Larbert, Forth Valley Royal Hospital: Norma Sidek; Leeds, St James Uni Hospital: William Cross, Peter Whelan; Leicester General Hospital: John Beatty; Leighton Hospital: Anna Tran, James Wylie; Lincoln County Hospital: Miguel Panades; London, Guy's Hospital: Sarah Rudman; London, North Middlesex Hospital: Nishi Gupta; London, Royal Free Hospital: Sarah Needleman, Maria Vilarino-Varela; London, UCH: Ursula McGovern; Macclesfield District General: James Dyer, Gerald Collins; Manchester, Salford Royal Hospital: Noel Clarke; Mayday Hospital/Croydon University Hospital: Angela Pathmanathan , Robert Huddart ; Mid Yorkshire Hospital: Rafal Turo, Subramanian Kanaga Sundaram; North Devon District Hospital: Mohini Varughese; Nottingham City Hospital: Santhanam Sundar, ; Portsmouth, Q Alexandra Hospital: Joanna Gale; Preston, Royal ~ Hospital: Alison Birtle; Reading, Royal Berks Hospital: Paul Rogers; Royal Berkshire Hospital: Helen O'Donnell; Royal Bolton Hospital: Ling Lee, Tony Elliott; Royal Glamorgan Hospital: Jacob Tanguay; Royal Marsden Hospital (London): Vincent Khoo; Royal Sussex County: Angus Robinson; Russells Hall Hospital: Pek Keng-Koh, Prakash Ramachandra, Mano Joseph; Scarborough Gen Hospital: Filip Kondylis, Andrew Robertson; Scunthorpe General: Sanjay Dixit; Southampton General Hospital: Simon Crabb; Southern General

Hospital/The New Victoria ACH: Abdulla Alhasso; St Leonards-on-Sea, Conquest Hospital: Caroline Manetta, Duncan Gilbert; St Mary's Hospital: Stephen Mangar, Simon Stewart ; Stepping Hill Hospital: James Dyer, Gerald Collins; Stevenage, Lister Hospital: Robert Hughes; Stockton-on-Tees, UH North Tees: John Logue; Sutton-in-Ashfield, King's Mill Hospital: Daniel Saunders, Georgina Walker; Swansea, Singleton: Ahmed Shaheen, Wael Mohamed; Tameside General Hospital: James Dyer, Gerald Collins; Taunton, Musgrove Park Hospital: Emma Gray, John Graham; Torbay District General Hospital: Anna Lydon; University Hospital Coventry and Warwickshire NHS Trust: Jane Worlding, Andrew Stockdale; University Hospital Wales: Krishna Narahari, Howard Kynaston; Weston General Hospital: Serena Hilman; Wexham Park Hospital: Nicola Dallas, Marc Laniado; Worcestershire Royal Hospital: Lisa Capaldi.

Supplementary Table 1: Baseline Characteristics by Trial Network and Randomization Ratio

	PATCH N=1082						STAMPEDE N=278		Total N=1360	
	1:2		1:1		Total					
	No.	%	No.	%	No.	%	No.	%	No.	%
**Treatment Arm**										
LHRHa	50	35%	449	48%	499	46%	140	50%	639	47%
tE2	94	65%	489	52%	583	54%	138	50%	721	53%
**Age**										
Median (IQR)	74.5 (70 – 78.5)		73 (68 - 78)		73 (68 – 78)		70 (64 – 75)		72 (68 – 77)	
Range	57 - 90		52 - 90		52 - 90		46 - 85		46 - 90	
<70	34	24%	303	32%	337	31%	126	45%	463	34%
>=70	110	76%	635	68%	745	69%	152	55%	897	66%
**WHO performance status**										
0: Normal activity without restriction	91	63%	699	75%	790	73%	242	87%	1,032	76%
1: Strenuous activity restricted, can do light work	43	30%	217	23%	260	24%	33	12%	293	22%
2: Up and about >50% of waking hours, limited self-care	10	7%	22	2%	32	3%	3	1%	35	3%
**Tumour status**										
T0	0	0%	0	0%	0	0%	1	0%	1	0%
T1	1	1%	8	1%	9	1%	1	0%	10	1%
T2	5	3%	28	3%	33	3%	13	5%	46	3%
T3	127	89%	806	86%	933	86%	224	81%	1,157	85%
T4	9	6%	94	10%	103	10%	36	13%	139	10%
Tx	1	1%	2	0%	3	0%	3	1%	6	0%
**Nodal status**										
N0	50	35%	656	70%	706	65%	177	64%	883	65%
N+	24	17%	190	20%	214	20%	100	36%	314	23%
NX	70	49%	92	10%	162	15%	1	0%	163	12%
**PSA**										
Median (IQR)	35.0		22.3		23.4 (11.5 – 51.6)		27.5 (12.6 – 61)		24.4 (11.8 – 54.1)	

	PATCH N=1082						STAMPEDE N=278		Total N=1360	
	1:2		1:1		Total					
	No.	%	No.	%	No.	%	No.	%	No.	%
	(18.1 – 73.2)		(10.7 – 47.9)							
Range	1.1 - 414		0.8 – 995		0.8 - 995		1.1 - 2488		0.8 - 2488	
<50	91	63%	714	76%	805	74%	188	68%	993	73%
50 – 499.9	53	37%	220	23%	273	25%	89	32%	362	27%
>=500	0	0%	4	0%	4	0%	1	0%	5	0%
**Gleason Sum Score**										
4	1	1%	0	0%	1	0%	0	0%	1	0%
5	1	1%	7	1%	8	1%	1	0%	9	1%
6	13	9%	55	6%	68	6%	0	0%	68	5%
7	59	41%	349	37%	408	38%	58	21%	466	34%
8	30	21%	188	20%	218	20%	79	28%	297	22%
9	38	27%	317	34%	355	33%	136	49%	491	36%
10	1	1%	17	2%	18	2%	5	2%	23	2%
**Gleason Sum Score (grouped)**										
4-6	15	10%	62	7%	77	7%	0	0%	77	6%
7	59	41%	349	37%	408	38%	58	21%	466	34%
8-10	69	48%	522	56%	591	55%	220	79%	811	60%
**RT to prostate planned as part of treatment?**										
No	132	92%	279	30%	411	38%	21	8%	432	32%
Yes	12	8%	659	70%	671	62%	257	92%	928	68%
**SOC docetaxel / abiraterone planned?**										
No	144	100%	922	98%	1,066	99%	213	77%	1,279	94%
Docetaxel	0	0%	16	2%	16	1%	64	23%	80	6%
Abiraterone	0	0%	0	0%	0	0%	1	0%	1	0%

Legend: From the PATCH network 1029/1082 (95%) were newly diagnosed at trial entry and 53/1082 (5%) were relapsing post radical therapy with a rising PSA. From the STAMPEDE network 266/278 (96%) were newly diagnosed and 12/278 (4%) were relapsing. Abbreviations: RT = radiotherapy, SOC = standard of care

Supplementary Table 2: Causes of Death

	LHRHa n=230		tE2 n=271		Total n=501	
	**N**	**%**	**N**	**%**	**N**	**%**
**Prostate Cancer**	92	40	125	46	217	43
**Other Malignancy**	32	14	33	12	65	13
**Cardiovascular**	34	15	26	10	60	12
**Respiratory**	34	15	45	17	79	16
**Infections***	14	6	14	5	28	6
**Gastrointestinal**	5	2	3	1	8	2
**Other**	14	6	15	6	29	6
**Missing**	5	2	10	4	15	3
**Total**	230	100%	271	100%	501	100%

Legend: Causes of death by treatment allocation

* Infections include 6 COVID deaths, 1 on LHRHa, 5 on tE2.

Note: One additional LHRHa patient had a cause of death but not a date of death therefore not included in the MFS analysis and not included in this table.

Supplementary Table 3: Estradiol at Different Time Points

		LHRHa	tE2	Total
Baseline	>250	6/495 (1%)	12/580 (2%)	18/1075 (2%)
	Mean (CI)	122 (117, 126)	121 (116, 127)	
1 month	>250	2/364 (1%)	486/507 (96%)	488/871 (56%)
	Mean (CI)	66 (61, 71)	1019 (957, 1080)	
3 months	>250	1/366 (<1%)	464/479 (97%)	465/845 (55%)
	Mean (CI)	58 (54, 62)	899 (844, 953)	
6 months	>250	1/393 (<1%)	456/472 (97%)	457/865 (53%)
	Mean (CI)	58 (54, 62)	954 (895, 1013)	
12 months	>250	1/316 (<1%)	321/333 (96%)	322/649 (50%)
	Mean (CI)	60 (56, 64)	975 (909, 1042)	

Legend: Number of patients with estradiol >250 pmol/L, for patients still on treatment. Mean levels and 95% confidence intervals (CI). Windows around timepoints: ± 1week at one month, ± 4 weeks at later points.

Supplementary Table 4

A: Sustained Castration Rates over one year by Target Testosterone Levels

**Analysis**	**LHRHa (N=490)**	**tE2 (N=576)**
T<1.7	85% (81%, 88%)	81% (77%, 84%)
T<1.7 + Estradiol >250	85% (81%, 88%)	85% (81%, 88%)
T<0.7	59% (54%, 63%)	47% (43%, 52%)
T<0.7 + Estradiol >250	59% (54%, 63%)	51% (47%, 56%)

Legend: T = Testosterone nmol/L, estradiol pmol/L

B: Castration Rates by Make of Patch Over Time

	LHRHa	tE2	FemSeven	Progynova
Month 1	60% (215/360)	83% (418/506)	81% (334/411)	88% (84/95)
Month 3	92% (343/371)	92% (441/478)	91% (352/388)	99% (89/90)
Month 6	93% (371/401)	91% (429/471)	90% (341/378)	95% (88/93)
Month 12	97% (317/327)	94% (311/331)	94% (252/268)	94% (59/63)

Legend: Number of patients recruited through the PATCH network of sites with castrate levels of testosterone (< 1.7nmol/L) at specific time points and by starting brand of patch for tE2 patients, for patients still on treatment. Windows around timepoints: ± 1week at one month, ± 4 weeks at later points.

Supplementary Figure 1: Overview of the tE2 Evaluation Program

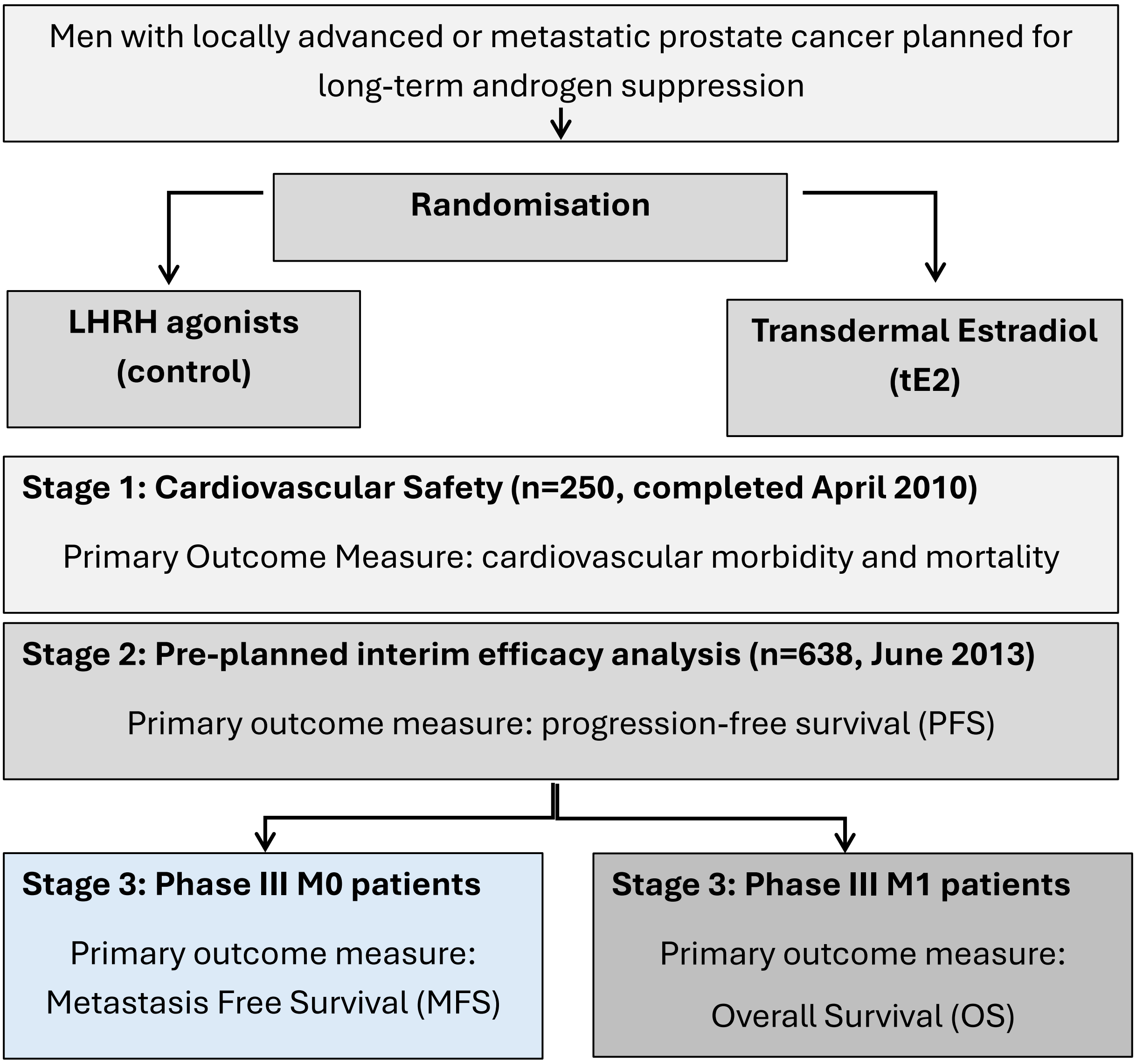

Legend Figure S1 – Summary of the tE2 Patch Repurposing Program

The program evolved from a phase II proof-of-concept trial into 2 individually powered phase III trials, one for patients with locally advanced disease (M0) and one for patients with metastatic disease (M1) at trial entry. Primary outcome events for the M1 trial are still accruing and will be reported separately. The pre-planned efficacy analysis was based on progression-free survival and reviewed by the Independent Data Monitoring Committee who then made recommendations about continuation of the evaluation. The purpose was to inform decisions about (and planning for) an extension into a phase III evaluation and to minimise any break in recruitment. Twenty eight percent (379/1360) of patients included in this report were included in the interim analysis and only 44/568 (8%) of the MFS events had occurred.

Supplementary Figure 2

A. Metastasis-Free Survival by Trial Network and PATCH Randomization Ratio with i) 90% and ii) 95% confidence intervals shown

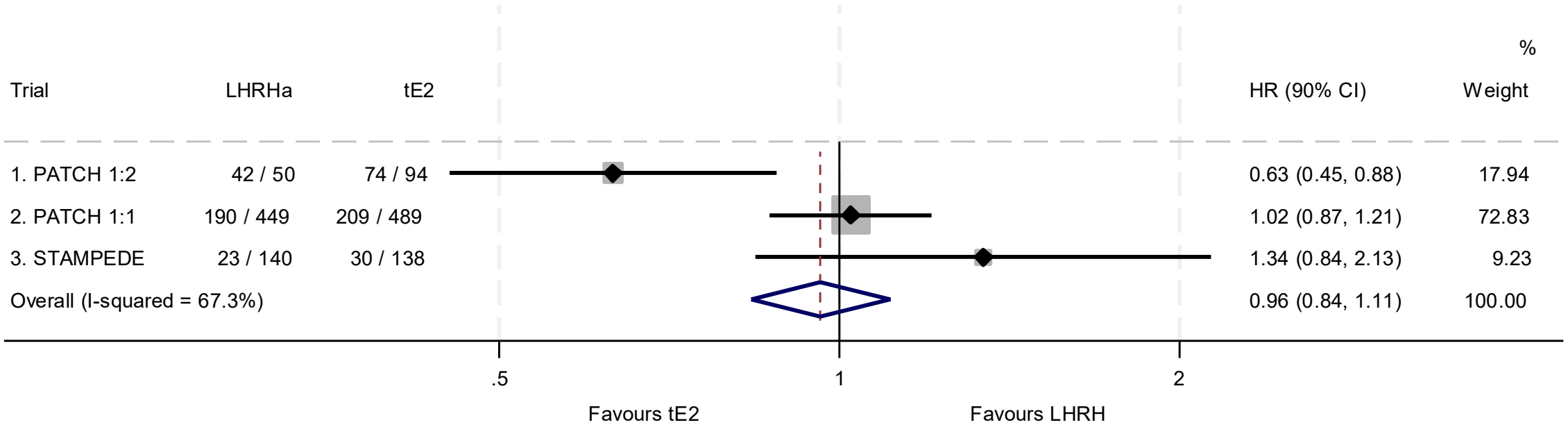

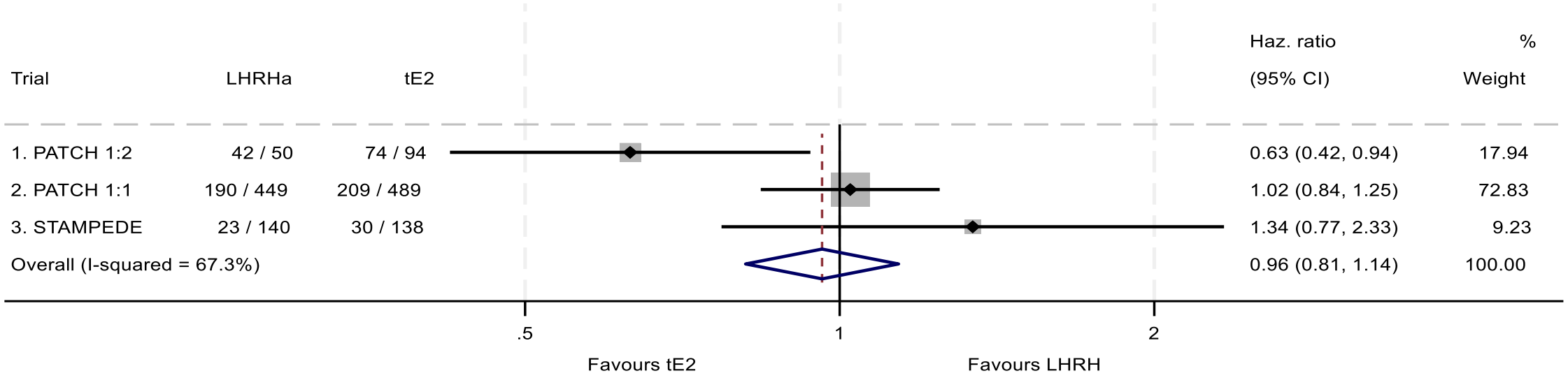

B. Metastasis-Free Survival – Effect of Prostate Radiotherapy

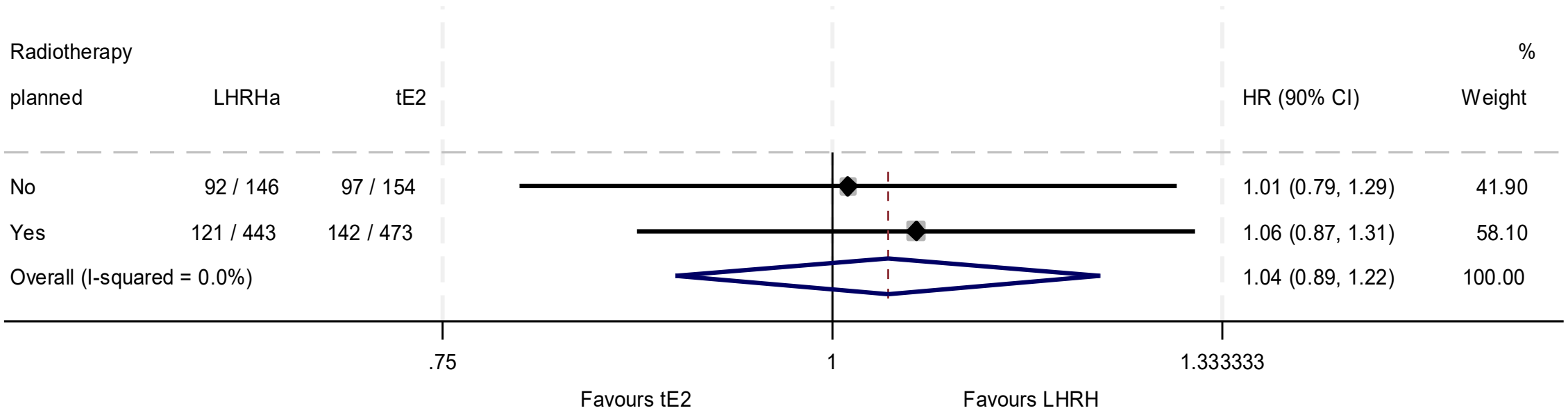

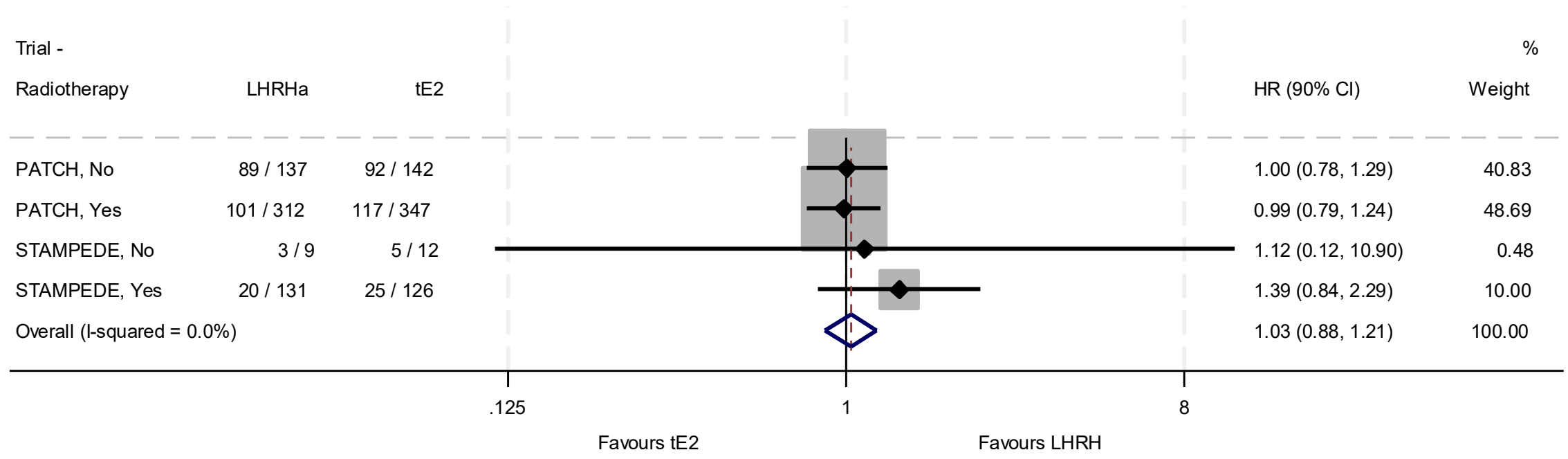

C. Overall Survival by Trial Network and PATCH Randomization Ratio

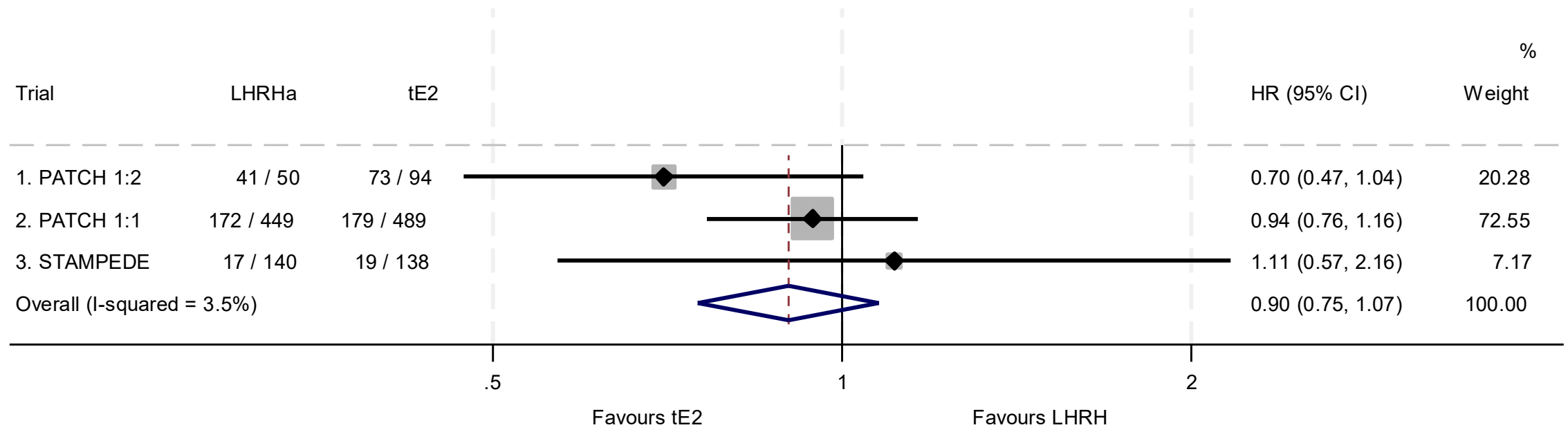

Legend: Panel A: As there was evidence of heterogeneity using the Cochran's Q test (p = 0.047) across the three trial cohorts the analysis for MFS was re-run as a sensitivity analysis using a random effects model giving a HR of 0.93 (90% CI 0.69-1.28) and still demonstrating non-inferiority. **Panel B:** Exploratory analyses to investigate the effect of prostate radiotherapy on MFS. The PATCH 2:1 cohort was excluded from this analysis as the participants were recruited when prostate radiotherapy was not standard of care. Plots show data combined from both trials and separately. In both analyses, the overall effect demonstrates non-inferiority of tE2 to LHRHa, and neither analysis shows any evidence of heterogeneity (Cochran's Q p-value of 0.794 and 0.781 for the first and second analyses respectively).

Supplementary Figure 3

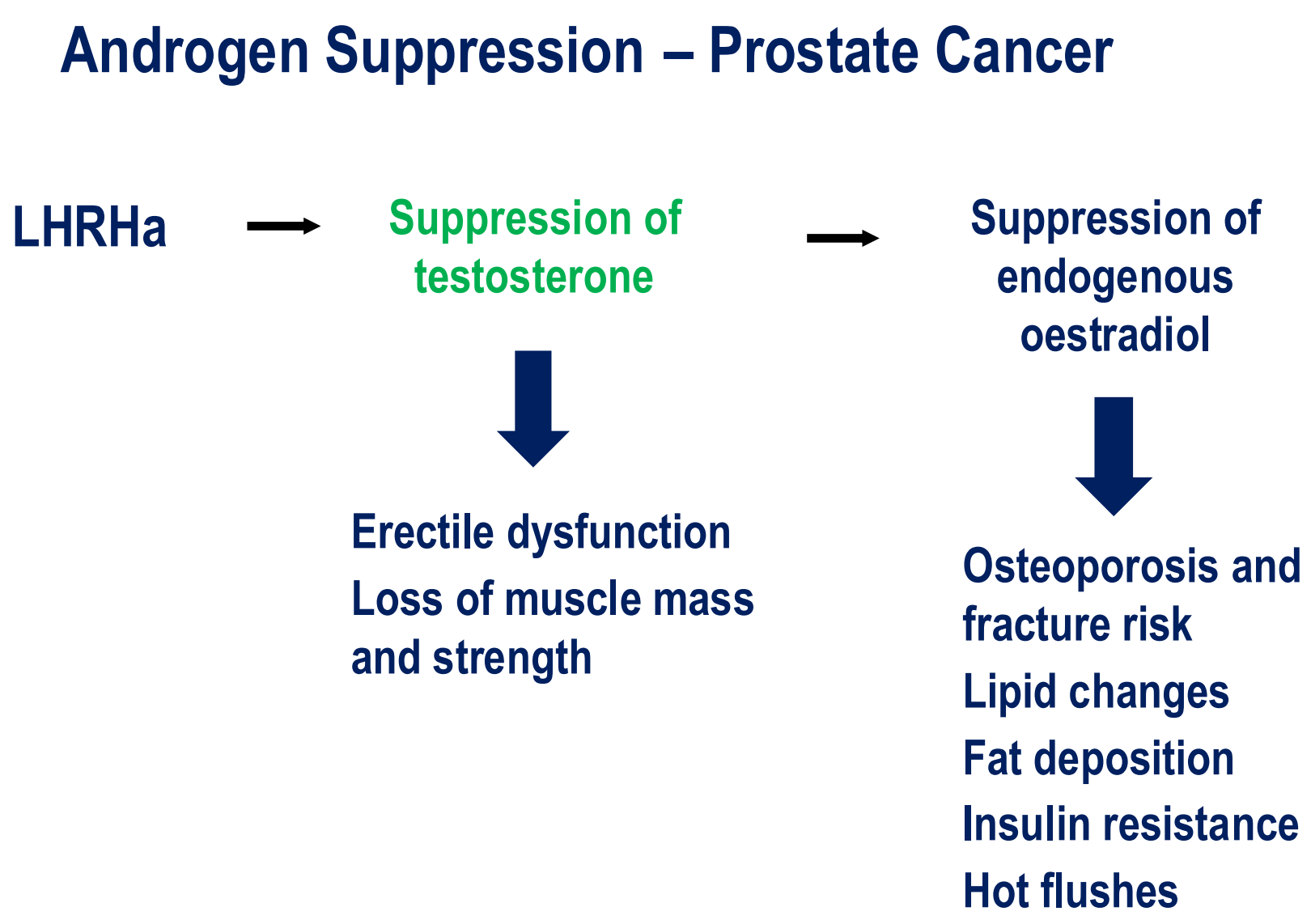

Legend: Androgen Deprivation Therapy with LHRHa: Hormone depletion and associated side-effects. For a comprehensive review see reference 4.

Additional Information

1. Further Statistical Details

As described in the statistical analysis plan, the meta-analysis used a two-stage approach, using a fixed effects model. Covariates for adjustment were decided prior to analysis, based on factors used to stratify randomization including age at randomisation, baseline PSA, intention to give radiotherapy, intention to give docetaxel, nodal status, WHO performance status, prior LHRH use, and allocation ratio. Overall estimates and confidence intervals were obtained from a Cox model, using the IPDmetan command in Stata. Proportional hazards assumptions were checked using the Cox models from each of the trials individually (PATCH 1:2, PATCH 1:1, STAMPEDE), with no evidence of non-proportionality (p-values 0.2222, 0.1511, 0.4483 respectively).

2. Exemplar Costs

The cost of an intervention is dependent upon the healthcare setting. UK British National Formulary data (bnf.nice.org.uk) accessed October 2025 indicate Femseven 100 mcg patch (3 patches changed twice weekly for 12 weeks) is £124.20 and are self-administered. A Zoladex LA 10.8 mg implant is £235.00 plus associated costs with administration.
